# Establishment and partial characterisation of a new cell line derived from adult tissues of the tsetse fly *Glossina morsitans morsitans*

**DOI:** 10.1186/s13071-024-06310-9

**Published:** 2024-05-17

**Authors:** Lesley Bell-Sakyi, Lee R. Haines, Giovanni Petrucci, Alexandra Beliavskaia, Catherine Hartley, Jing Jing Khoo, Benjamin L. Makepeace, Adly M. M. Abd-Alla, Alistair C. Darby

**Affiliations:** 1 Department of Infection Biology and Microbiomes, Institute of Infection, Veterinary and Ecological Sciences, University of Liverpool, Liverpool, UK; 2 Vector Biology Department, Liverpool School of Tropical Medicine, Liverpool, UK; 3 Insect Pest Control Laboratory, Joint FAO/IAEA Centre of Nuclear Techniques in Food and Agriculture, Vienna, Austria; 4 Present Address: Department of Biological Sciences, University of Notre Dame, South Bend, IN USA

**Keywords:** insect, cell line, tsetse, *Glossina morsitans morsitans*, iflavirus, negevirus, *Wolbachia*

## Abstract

**Background:**

Insect cell lines play a vital role in many aspects of research on disease vectors and agricultural pests. The tsetse fly *Glossina morsitans morsitans* is an important vector of salivarian trypanosomes in sub-Saharan Africa and, as such, is a major constraint on human health and agricultural development in the region.

**Methods:**

Here, we report establishment and partial characterisation of a cell line, GMA/LULS61, derived from tissues of adult female *G. m. morsitans*. GMA/LULS61 cells, grown at 28 °C in L-15 (Leibovitz) medium supplemented with foetal bovine serum and tryptose phosphate broth, have been taken through 23 passages to date and can be split 1:1 at 2-week intervals. Karyotyping at passage 17 revealed a predominantly haploid chromosome complement. Species origin and absence of contaminating bacteria were confirmed by PCR amplification and sequencing of fragments of the COI gene and pan-bacterial 16S rRNA gene respectively. However, PCR screening of RNA extracted from GMA/LULS61 cells confirmed presence of the recently described Glossina morsitans morsitans iflavirus and Glossina morsitans morsitans negevirus, but absence of *Glossina pallipides* salivary gland hypertrophy virus. GMA/LULS61 cells supported infection and growth of 6/7 different insect-derived strains of the intracellular bacterial symbiont *Wolbachia*.

**Conclusions:**

The GMA/LULS61 cell line has potential for application in a variety of studies investigating the biology of *G. m. morsitans* and its associated pathogenic and symbiotic microorganisms.

**Graphical Abstract:**

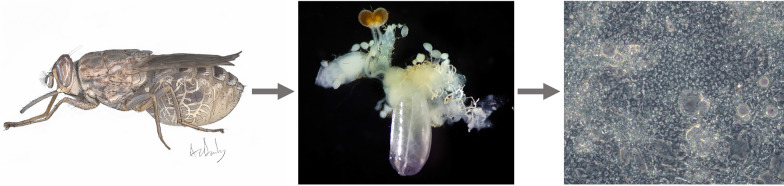

**Supplementary Information:**

The online version contains supplementary material available at 10.1186/s13071-024-06310-9.

## Background

Tsetse flies (Diptera: Glossinidae) are vectors of several species of salivarian trypanosomes, protozoan parasites of humans and domestic animals in sub-Saharan Africa [1, 2], and also harbour a range of bacterial symbionts including *Wolbachia* [3, 4]. Research at the cellular and molecular level into biology and control of tsetse flies, and their interactions with bacterial symbionts and pathogenic trypanosomes, would be greatly assisted by the use of cell lines derived from tsetse tissues. A cell line derived from larval *Glossina morsitans* (subspecies not specified) was reported over 45 years ago [5, 6]. This cell line, designated WR73-Gm-1 [6], was grown in modified Schneider’s *Drosophila* medium supplemented with 15–20% foetal bovine serum (FBS) at 25 °C and comprised a monolayer of epithelial-like [6] or spindle-shaped and epithelial-like cells [5]. Co-cultivation of bloodstream forms of *Trypanosoma congolense* with the tsetse cells resulted in transformation of the parasites to insect stages, continuous growth of procyclic forms and differentiation into epimastigotes [5]. The WR73-Gm-1 cell line survived for at least 6 years and underwent at least 43 subcultures [7]. However, to our knowledge, no further reports of application of this larva-derived tsetse cell line were published [8] and its fate remains unknown.

One of the key functions of the Tick Cell Biobank, recognised as the world’s only dedicated culture collection for cell lines derived from ticks and other arthropods, is to generate new cell lines from arthropods of medical and veterinary importance and make them available as essential research tools [9]. Considering the importance of tsetse flies as vectors of human and animal diseases, and the current non-availability of any tsetse cell line, we initiated attempts to generate primary cultures of larval *G. m. morsitans* with a view to cell line establishment. While we did not obtain any long-lasting larval cell cultures, we succeeded in generating a cell line from adult female tissues. Here, we describe the establishment of this cell line, designated GMA/LULS61, present the results of screening the cells for viruses of potential importance as tools for tsetse control,and report their susceptibility to infection with a panel of strains of the intracellular bacterial symbiont *Wolbachia*.

## Methods

### Tsetse flies

The *G. m. morsitans* Westwood colony, originally derived from pupae collected in Zimbabwe, was established at the Liverpool School of Tropical Medicine in 2004. Flies were reared at 26 ± 2 °C, 68–76% relative humidity, with a 12-h photoperiod. Adult flies were fed three times per week on defibrinated horse blood (TCS Biosciences, Buckingham, UK) using artificial silicone feeding membranes [10].

### Generation of primary cell cultures

**Fig. 1:**
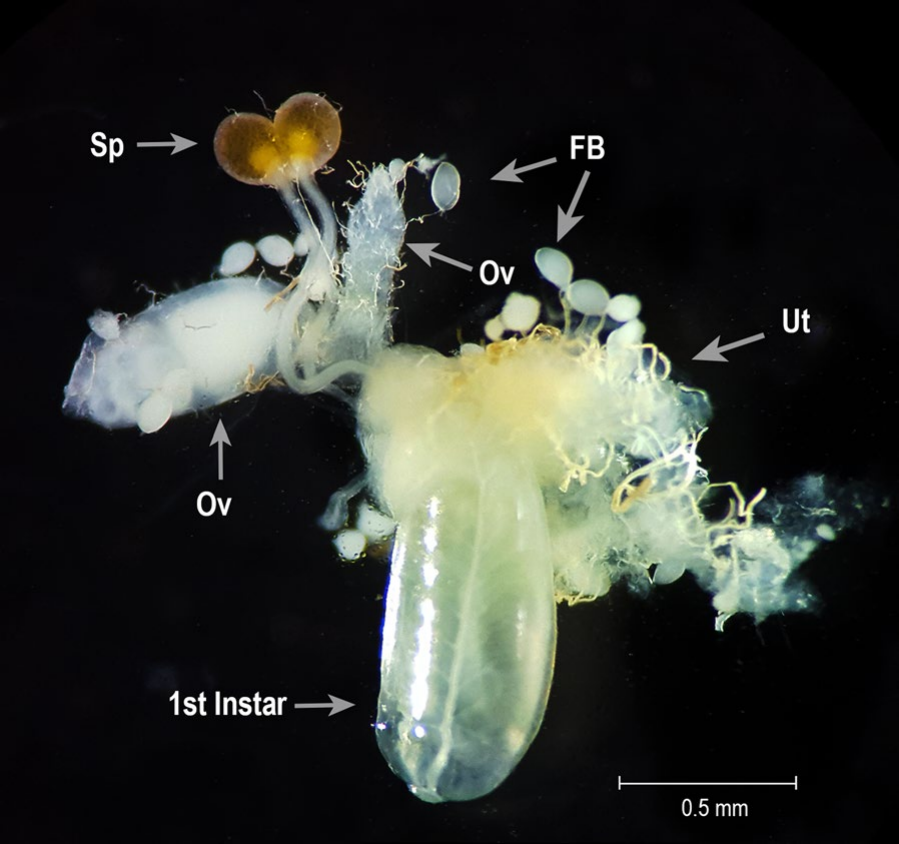
Reproductive tissues dissected out from the body of an adult female *Glossina morsitans morsitans* fly. The uterus was gently retracted to visualise the posterior end of the developing larva. *Ov* ovarioles, *Sp* spermatheca, *FB* fat body, *Ut* uterus, *1st instar* L1 larva. Scale bar = 0.5 mm

On 28 June 2018, 10-week-old female *G. m. morsitans* with large white abdomens (typically indicative of pregnancy) were cold-anaesthetised in a Petri dish on ice. Flies had received their last feed of defibrinated horse blood 5 days earlier to minimise blood meal contamination during dissection. Using dissecting tweezers, each fly’s head was pinched off at the neck, and the headless body was transferred into a class II microbiological safety cabinet where it was surface-sterilised by immersion in either 70% ethanol for 1–2 min or 0.1% benzalkonium chloride for 1 min, followed by 70% ethanol for 1 min, and placed on sterile absorbent paper towel for 1 min to remove excess fluid. The body was then dissected aseptically in a 20-µl drop of sterile phosphate-buffered saline on a sterile glass slide using sterile watchmakers’ forceps. The penultimate abdominal segment was grasped, and the reproductive tissues were gently pulled out of the posterior end of the abdomen to avoid midgut rupture. The developing ovarioles, spermathecae, uterus and larva were freed from other connective tissues (Fig. 1), and the larva was transferred to Hanks balanced salt solution (HBSS) in a sterile bijou container. The developmental stage of each larva was recorded as instar stage L1, L2 or L3.

Within 2 h of harvest, each larva was individually macerated using sterile watchmakers’ forceps and the tissue pieces transferred to a flat-sided cell culture tube (Nunc, Thermo-Fisher, Loughborough, UK) with 2 ml complete culture medium. Membrane-like tissues of adult origin surrounding four L1 larvae and two fertilised eggs (isolated from the uterus) were collected and placed together in a separate culture tube with medium. Four complete culture media were used: L-15 (Leibovitz) supplemented with 10% tryptose phosphate broth (TPB) (L-15), L-15B [11] supplemented with 10% TPB and 0.1% bovine lipoprotein concentrate (L-15B), HBSS supplemented with 0.5% lactalbumin hydrolysate (H-Lac) and Schneider’s *Drosophila* medium (Schneider’s). All media were additionally supplemented with 20% foetal bovine serum, 2 mM L-glutamine, 100 units/ml penicillin and 100 µg/ml streptomycin. Medium components were supplied by Invitrogen (Thermo-Fisher, Loughborough, UK) or Sigma (Sigma-Aldrich, Gillingham, UK). All cultures were incubated sealed, in ambient air, in a dry incubator at 28 °C. Medium was changed weekly by removal and replacement of ¾ of the volume using freshly prepared complete medium, and cultures were examined weekly by inverted microscope for evidence of cell growth.

### Development of the GMA/LULS61 cell line

When growing cells covered at least 50% of the flat surface of the culture tube, they were initially resuspended by gentle pipetting and allowed to reattach. Following regrowth to at least 75% confluence, subculture was carried out by adding 2.2 ml fresh complete medium, pipetting and transferring half of the resultant cell suspension to a new flat-sided tube. Later subcultures were made into T25 flasks by combining half of the cells from two flat-sided tubes to give a final volume of 5 ml. Subcultures from T25 flasks were subsequently made by addition of 5 ml fresh medium, resuspending the cells by scraping or pipetting and transferring 5 ml cell suspension to a new flask.

Cytocentrifuge smears of resuspended cells were periodically prepared using a Shandon Cytospin 4, air dried, fixed in methanol, stained with Giemsa and examined at × 1,000 magnification (oil immersion) using a transmission light microscope. Measurements of growing cells were carried out using a Zeiss Axiovert microscope with Zen Pro software (Carl Zeiss Ltd., Cambridge, UK). Supernatant medium was periodically screened for mycoplasma using at least two commercial kits as described previously [12]. Cells were cryopreserved with 10% dimethyl sulphoxide in vapour phase liquid nitrogen and resuscitated as described previously [13].

Metaphase chromosome spreads were prepared from GMA/LULS61 cells at passage 17 by a modification of a previously described method [13]. Briefly, a 67-day-old culture at passage 16 was resuspended by vigorous pipetting and divided between two daughter flasks. On day 3 after passage, the medium was changed, 2 ml of colcemid (10 µg/ml, Roche Diagnostics, Newhaven, UK) was added to the daughter flasks, and the cells were incubated overnight at 28 °C. On the next day, the medium was removed, and the cells were rinsed with PBS and incubated in 1 ml of 0.025% trypsin/0.01% EDTA (Invitrogen) for 5 min at 28 °C. Complete L-15 medium (3 ml) was added, the cells were harvested by scraping, centrifuged at 500 × g for 5 min, resuspended in 5 ml 0.7% sodium citrate and incubated for 35 min at 37 °C. The cells were centrifuged as before; the pellet was resuspended in 2.5 ml ice-cold acetic alcohol (3 parts methanol, 1 part glacial acetic acid) and held on ice for 5 min. This fixation step was repeated once, and then the cell pellet was resuspended in an equal volume of ice-cold acetic alcohol and deposited dropwise onto wet, ice-cold microscope slides from a height of 1 m. The slides were air-dried, stained in 3% Giemsa for 1 h and examined at × 300 magnification to locate countable metaphase chromosome spreads. The chromosomes were counted in 100 spreads.

### Molecular characterisation

GMA/LULS61 cells were resuspended by pipetting or scraping and centrifuged at 200 × g for 5 min at room temperature. DNA was extracted from cell pellets using a DNeasy Blood and Tissue Kit (Qiagen, Hilden, Germany) following the manufacturer’s instructions for cultured cells. To confirm species origin, PCRs targeting a ~ 1500 bp fragment of the eukaryotic 18S rRNA gene and a ~ 700 bp fragment of the mitochondrial COI gene were carried out as described previously [12, 14, 15]. To screen for contaminating bacteria, a pan-bacterial PCR targeting a fragment of the bacterial 16S rRNA gene [16] was performed as described previously [12]. Positive PCR products were detected by agarose gel electrophoresis, purified using a PureLink PCR purification kit (Thermo-Fisher, Loughborough, UK) and Sanger sequenced in both directions (Source Bioscience, Nottingham, UK). The resultant sequences were compared to published sequences in GenBank using BLAST. Phylogenetic trees were generated using the neighbour-joining Tamura-Nei model with 1000 bootstraps.

### Virus screening

GMA/LULS61 cells at passage 14 in a T25 flask were harvested by scraping, pelleted by centrifugation at 200 × g for 5 min, resuspended in 1 ml PBS, transferred to a 1.5 ml microfuge tube, centrifuged as before, and the cell pellet was overlain with 1.4 ml RNAlater^®^ (Sigma-Aldrich, Gillingham, UK). The cell pellet was then shipped from the Tick Cell Biobank to the Insect Pest Control Laboratory (IPCL) of the Joint FAO/IAEA Centre of Nuclear Techniques in Food and Agriculture, Seibersdorf, Austria, for subsequent processing. DNA and RNA were extracted using a TRIzol^™^ Reagent kit (Invitrogen, Thermo-Fisher Scientific, Waltham, MA, USA), and RNA was converted to cDNA using a Superscript III kit (Invitrogen, Thermo-Fisher Scientific, Waltham, MA, USA), following the manufacturer’s instructions. PCR reactions were conducted using the Platinum II master mix (Invitrogen, Thermo-Fisher Scientific, Waltham, MA, USA), while qPCR reactions were conducted with the iQ SYBR Green Supermix (Biorad Laboratories, Hercules, CA, USA). Primers targeting a specific sequence of the salivary gland hypertrophy virus (SGHV) of *Glossina pallipides* [17], the RNA-dependent RNA polymerase (RdRp) gene of Glossina morsitans morsitans iflavirus (GmmIV), the RdRp gene of Glossina morsitans morsitans negevirus (GmmNegeV) [18] and PCR conditions are presented in Table 1. Primers targeting tsetse tubulin were used as a control for the nucleic acid extractions, and DNA from the oriental fruit fly *Bactrocera dorsalis* was used as a negative control. All reactions included a no-template control. Positive PCR products were detected by agarose gel electrophoresis, purified using a DNA Clean & Concentrator Kit (Zymoresearch, Freiburg im Breisgau, Germany) and Sanger sequenced in both directions (Eurofins Genomics, Ebersberg, Germany). The resultant sequences were compared to published sequences in GenBank using BLAST.

**Table 1:** Primers and PCR conditions for detection and quantification of viruses in *Glossina morsitans morsitans* cell line GMA/LULS61

Target	Primer name	Primer sequence	Annealing temperature °C	Expected amplicon size (bp)	Reference
Primers for RT-PCR and PCR					
GmmIV	Iflav-tseCont1-1F	TTTGCCTTTGTCCTTTAGATGTGCT	58	542	[18]
	Iflav-tseCont1-1R	AAATGGCTACGCGATGTAGAATGG			
GmmNV	Nege-PCR_2256F	CCGATGGTCATTGTACCGAATTGCGTCCTAAGT	58	535	This study
	Nege-PCR_2791R	CATAACGGCAGCGTCACTCATAAC			
GpSGHV	GpSGHV2F	CTTGTCAGCGCCACGTACAT	58	401	[40]
	GpSGHV2R	GCATTCACAGCATCCCAATTTT			
Tubulin	Tsetse-tubulinF	GATGGTCAAGTGCGATCCT	58	355	[41]
	Tsetse-tubulinR	TGAGAACTCGCCTTCTTCC			
Primers for qRT-PCR and qPCR					
GmmIV	Ifla_qPCR2_7848F	AGAAATTGAAGGACAGATGTTTGGT	62	99	[18]
	Ifla_qPCR2_7947R	ACCTAAGAAATTACCAGTACCCTCC			
GmmNV	Nege_qPCR1-2411F	CAACATAGACTTGAACCAGAGCA	62	118	[18]
	Nege_qPCR1-2529R	GAAACATCAAACACACTCCCATTAG			
GpSGHV	QPCRFwd	CAAATGATCCGTCGTGGTAGAA	60	51	[17]
	QPCRRev	AAGCCGATTATGTCATGGAAGG			
Tubulin	Tsetse-tubulinF	GATGGTCAAGTGCGATCCT	62	355	[41]

### Infection of GMA/LULS61 cells with *Wolbachia*

GMA/LULS61 cells were seeded in flat-sided tubes in 2.2 ml complete L-15 medium and incubated at 28 °C for 7 days. A panel of seven strains of *Wolbachia* were grown in insect cell lines as summarised in Table 2. Culture supernatant containing cell-free *Wolbachia* of each test strain was passed through a 0.45-µm filter to remove any host cells, 0.3–0.5 ml of the filtrate was inoculated into GMA/LULS61 cells and the tubes were returned to 28 °C. Medium was changed weekly and cultures were examined by inverted microscope for signs of cytopathic effect (CPE). Giemsa-stained cytocentrifuge smears were prepared and examined for presence of *Wolbachia*-infected cells after 3 weeks and at intervals thereafter up to 13 weeks. DNA was extracted from cultures visually negative for intracellular bacteria as described in Sect. 2.4 and screened for presence of *Wolbachia* using a qPCR targeting a 99-bp fragment of the 16S rRNA gene as described previously [19].

**Table 2:** Origins and laboratory host cell lines used in the present study to culture *Wolbachia* strains tested for infectivity for GMA/LULS61 cells

*Wolbachia* strain	Natural host	References	Laboratory host cell line (reference)
*w*Pip	*Culex pipiens molestus*	[12]	*C. p. molestus* CPL/LULS56 [12]
*w*Pap	*Phlebotomus papatasi*	[12]	*P. papatasi* PPL/LULS49 [12]
*w*CfeJ	*Ctenocephalides felis*	[42, 43]	*Ixodes scapularis* IDE8 [44]
*w*AlbB	*Aedes albopictus*	[45]	*Ae. albopictus* C6/36^1^ [46]
*w*Bol1-b	*Hypolimnas bolina*	[47]	*Spodoptera frugiperda* Sf9^2^ [48]
*w*Str1	*Laodelphax striatellus*	[49]	*S. frugiperda* Sf9^3^ [48]
*w*MelPop-CLA	*Drosophila melanogaster*	[50]	*Ae. albopictus* RML-12 [50]

^1^Transferred from *Ae. albopictus* cell line Aa23 [45].

^2^Transferred from RML-12 [47].

^3^Transferred from *Ae. albopictus* cell line C7-10[51]

## Results

Eight primary *G. m. morsitans* cell cultures were initiated in four different complete culture media in June 2018. Of these, seven were generated from tissues of larvae at stages L1 (*n* = 1), L2 (*n* = 3) or L3 (*n* = 3), and one was generated from the adult female membranous tissue surrounding the stage 1 larvae (Table 3). Although some tissues in the larva-derived cultures had attached by day 4, and some of these exhibited contractions over the subsequent 5 months, none of them exhibited significant cell growth. After 6 months, the two cultures in Schneider’s medium were combined and the four cultures in L-15B and H-Lac were combined in an attempt to stimulate growth by increasing the volume of tissues per culture. These two combined cultures derived from stage 2 and 3 larvae ceased to metabolise and were discarded after 8 months, and the culture derived from stage 1 larvae was discarded after 18 months.

**Table 3:** Summary of attempts to generate primary *Glossina morsitans morsitans* cell cultures in the present study

Culture	Tissue (stage)	Medium^1^	Outcome
1	Larva (L3)	L-15	Metabolism but no growth, combined with #2, 4 and 6 on day 189, discarded on day 252
2	Larva (L3)	L-15B	Metabolism but no growth, combined with #1, 4 and 6 on day 189, discarded on day 252
3	Larva (L3)	Schneider’s	Small patch of growing cells seen on day 79, three patches seen on day 99; combined with #5 on day 189, no further growth, discarded on day 252
4	Larva (L2)	H-Lac	Metabolism but no growth, combined with #1, 2, and 6 on day 189, discarded on day 252
5	Larva (L2)	Schneider’s	Some contracting cells seen on day 79, twitching cell clumps seen on day 125, combined with #3 on day 189, no further growth, discarded on day 252
6	Larva (L2)	L-15B	Metabolism but no growth, combined with #1, 4 and 6 on day 189, discarded on day 252
7	Two larvae (L1)	L-15, L-15B from day 7	Metabolism but no growth, discarded on day 562
8	Adult tissues	L-15	Slow growth, 1st passage on day 437. Developed into cell line GMA/LULS61

^1^Medium formulations are described in Methods

**Fig. 2:**
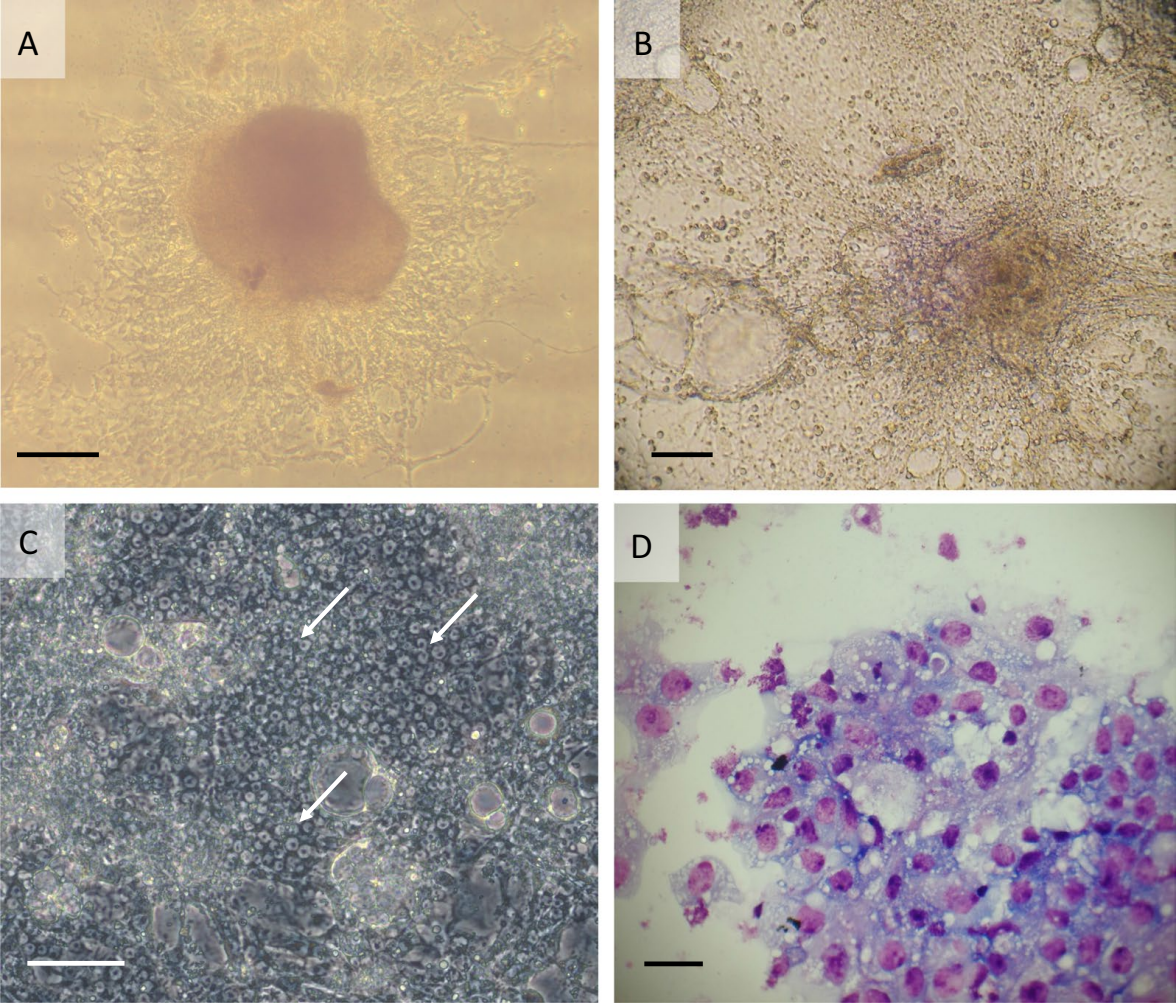
*Glossina morsitans morsitans* adult-derived cell line GMA/LULS61. **A** Primary culture at 14 months after initiation; live, phase contrast inverted microscope. **B** GMA/LULS61 cells at passage 4, 18 months after initiation; live, inverted microscope. **C** GMA/LULS61 cells at passage 23, 65 months after initiation showing areas of identifiable individual cells (arrows); live, phase contrast inverted microscope. **D** Giemsa-stained cytocentrifuge smear of GMA/LULS61 cells at passage 23. Scale bars = 100 µm (**A**, **B**), 50 µm (**C**), 10 µm (**D**)

The primary culture initiated in complete L-15 from adult tissues followed a different path, ultimately developing into the cell line GMA/LULS61. Contracting tissues were present in the primary culture between days 4 and 42. A small area of attached tissue slowly increased in size, although at first it was unclear whether this was due to cell migration or actual multiplication. On day 163 the attached cells were reseeded by gentle pipetting, and subsequently it became evident that they were growing as each small reattached clump expanded outwards (Fig. 2A). By day 437, at least 75% of the flat surface of the tube was covered with cells, and the first subculture was carried out. Additional subcultures were made from the primary culture on days 474 and 500; the first subculture was taken to passage 2 on day 519 (~ 17 months after initiation). The cells were transferred from flat-sided tubes into T25 flasks at 22 months (passage 3). Cells were passaged initially at 8-week intervals; from passage ~ 12 it became possible to split the cells at 4–6-week intervals and from passage ~ 20 at 2–3-week intervals. The first attempt to cryopreserve the cells at 28 months (passage 2) was unsuccessful; small cell clumps survived but did not grow over the subsequent 11 weeks and the culture was discarded. A second attempt at 41 months (passage 6) was successful, with cells starting to grow within a week of resuscitation. At the time of writing, nearly 6 years after initiation, the GMA/LULS61 cell line has reached passage 23. Annual screening for contamination with *Mycoplasma* has consistently yielded negative results, and absence of contaminating bacteria was confirmed by failure to amplify any product using the pan-bacterial PCR on DNA extracted from cells at passage 2.

The GMA/LULS61 cells grow in sheets forming an uneven monolayer. When detached by pipetting, scraping or enzymatic dissociation using trypsin/EDTA, they do not form a single cell suspension, but rather a suspension of variously sized clumps of cells with a large amount of debris. In the daughter culture, these cell clumps reattach within 24 h and cells begin to migrate out from them within a few days. A complete monolayer forms within 2–4 weeks, comprising sheets of single cell thickness, multicellular clumps with string-like multicellular projections down to the flask surface and, in older cultures, large bubble-like vesicles (Fig. 2B). Where it is possible to distinguish the borders of individual cells (Fig. 2C), the cells range between 7.0 and 9.3 µm in diameter (mean 7.91 µm). In Giemsa-stained cytocentrifuge smears, the cells appear as predominantly small, pleomorphic fibroblast-like or epithelial-like cells with vacuolated cytoplasm (Fig. 2D).

**Fig. 3:**
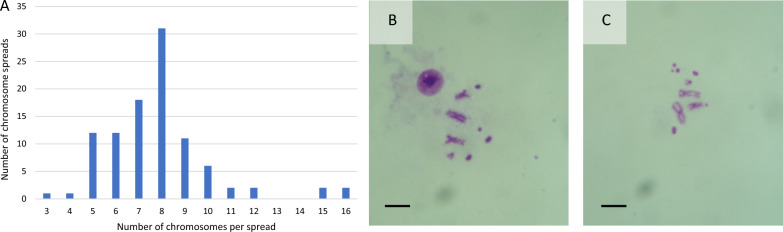
Karyotyping of *Glossina morsitans morsitans* cell line GMA/LULS61 at passage 17. **A** Distribution of chromosome numbers in 100 metaphase spreads. **B** Typical spread with the modal number of 8 chromosomes. **C** Spread with 10 chromosomes. Scale bar = 5 µm

Karyotyping of a passage 17 culture revealed a modal number of 8 chromosomes per metaphase spread, with a range of 3–16 (Fig. 3A). Following the description of Southern and co-workers [20], in the majority of spreads in which chromosome morphology could be distinguished, there were two large approximately metacentric autosomes, a medium-sized sub-metacentric X chromosome and two or three small telocentric autosomes or “S” chromosomes. In addition, many spreads contained one or more putative microchromosomes or chromosome fragments (Fig. 3B, C). Notably, spreads with more than two large metacentric autosomes were rarely seen.

**Fig. 4:**
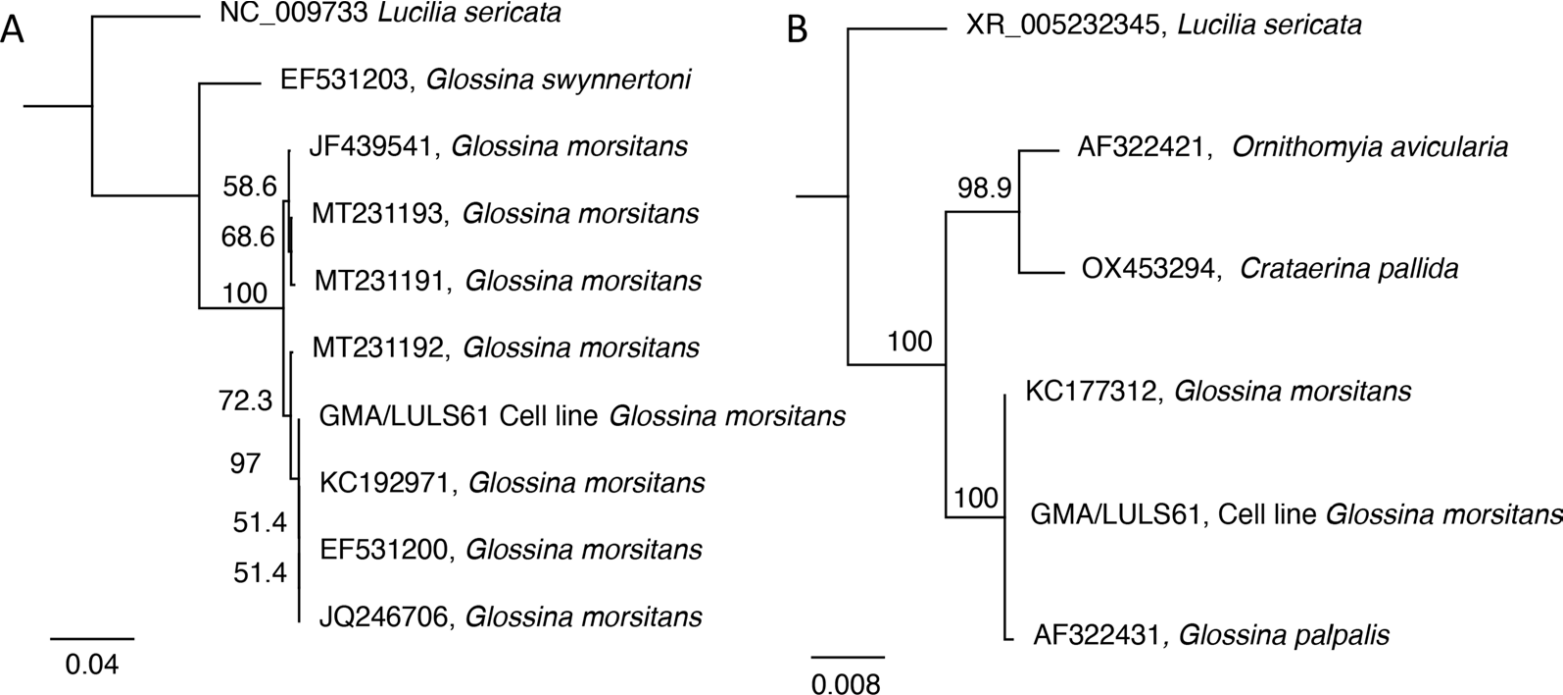
Phylogenetic analysis of sequences amplified from DNA extracted from the *Glossina morsitans morsitans* cell line GMA/LULS61. **A** The mitochondrially encoded cytochrome c oxidase I (COI) gene. **B** 18S ribosomal DNA (rDNA). Trees were generated using the neighbour-joining method with the Tamura-Nei model and bootstrapped 1000 times. Node labels show bootstrap support as a percentage

Species origin of the GMA/LULS61 cell line was confirmed by PCR amplification and sequencing of fragments of two genes from DNA extracted from cells at passage 2, 2 years after initiation of the primary culture. The 663-bp sequence obtained from the COI PCR was 100% identical to three published sequences from *G. morsitans* (KC192971, EF531200 and JQ246706, 100% query cover) and 99.25% identical to a fourth *G. morsitans* sequence (JF439541.1, 100% query cover). The 1111-bp sequence obtained from the eukaryotic 18S rRNA gene PCR was 99.91% identical to a published sequence from *G. morsitans* (KC177312.1, 100% query cover) and 99.46% identical to a published sequence from *Glossina palpalis* (AF322431.1, 100% query cover). Original geographic locations of the *Glossina* spp. from which the published sequences were derived were not stated in Genbank. Phylogenetic trees, generated from the two sequences (Fig. 4), place the GMA/LULS61 cell line firmly within the genus *Glossina* and the *G. morsitans* species complex. The sequences amplified from GMA/LULS61 DNA were deposited in NCBI GenBank under accession numbers PP348260 (COI) and PP348259 (18S rRNA).

**Fig. 5:**
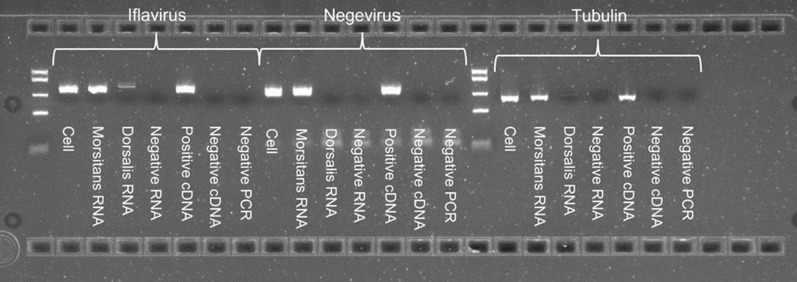
PCR screening of *Glossina morsitans morsitans* cell line GMA/LULS61 for presence of GmmIV and GmmNegeV. PCR products were analysed using 2% gel agarose stained with ethidium bromide and fastRuler low range DNA ladder (Thermo-Fisher Scientific, Waltham, MA, USA) with a band range of 50–1500 bp. Cell = GMA/LULS61 cell line. Morsitans = RNA extracted from *G. m. morsitans* adult known to be infected with GmmIV and GmmNegeV Dorsalis = RNA extracted from *Bactrocera dorsalis* adults. Negative RNA = negative control for the RNA extraction in which all RNA extraction procedures were performed without biological samples. Positive cDNA = cDNA from a previously RNA extraction proved positive for both GmmIV and GmmNegeV. Negative cDNA = cDNA from a previous RNA extraction proved negative for both GmmIV and GmmNegeV. Negative PCR = no cDNA template control

At passage 14 (57 months after initiation), GMA/LULS61 cells were screened for presence of RNA from two recently described *G. m. morsitans* RNA viruses, GmmIV and GmmNegeV. Quantitative PCRs were conducted targeting specific fragments of 99 and 118 bp for GmmIV and GmmNegeV, respectively. These PCRs revealed specific amplification with melting curves resembling these of the positive control samples (Additional file 1: Fig. S1). Additionally, standard PCRs targeting 542- and 535-bp fragments specific to the iflavirus and negevirus respectively yielded positive results for cDNA generated from RNA extracted from the GMA/LULS61 cells (Fig. 5). Sanger sequencing of the PCR products revealed that 506 nt from the iflavirus sequence matched to the GmmIV genome (accession number OL353434.1) with 99% identity. Furthermore, 435 nt of the negevirus sequence matched with the GmmNegeV genome (accession number OL353435.1) with 97% identity. Blastx analysis of the nucleotide sequence revealed a 100% identity match of the iflavirus sequence from the GMA/LULS61 cells with the polyprotein of GmmIV (accession number UGC12015.1). Additionally, the negevirus sequence matched with 99% identity to the RdRp of GmmNegeV (accession number UGC12016.1. A PCR targeting a 401-bp fragment of the occlusion-derived virus (*odv-e66*) gene of SGHV failed to amplify any product from DNA extracted from the GMA/LULS61 cells (data not shown). The viral sequences amplified from GMA/LULS61 RNA were deposited in GenBank under accession numbers PP405073 (GmmIV) and PP405074 (GmmNegeV).

**Fig. 6:**
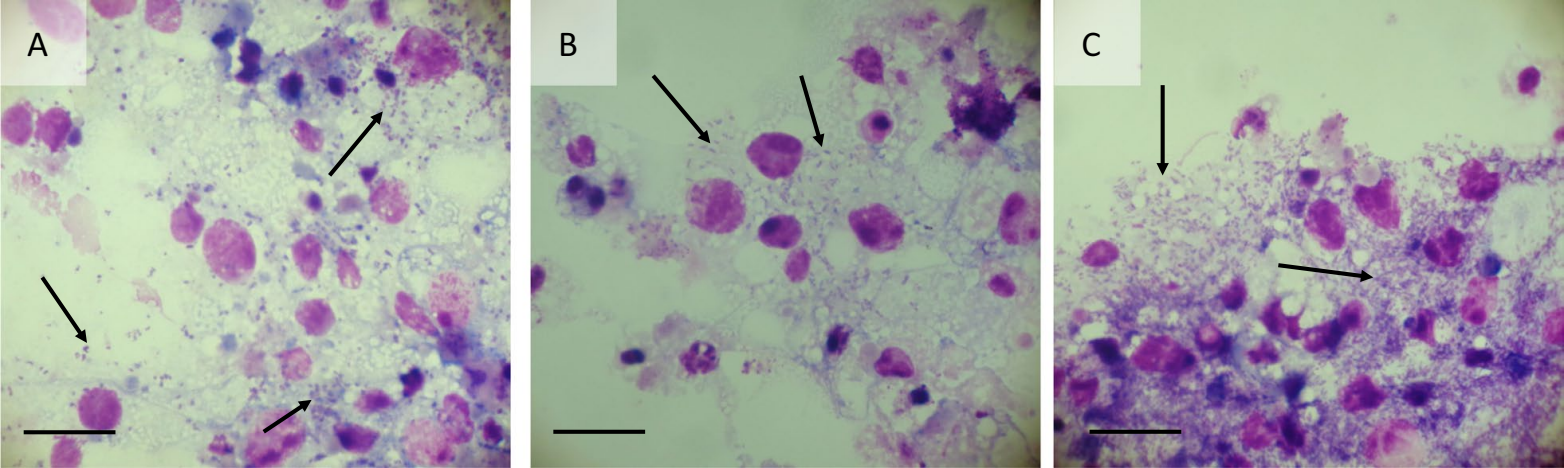
Giemsa-stained cytocentrifuge smears of *Wolbachia*-infected GMA/LULS61 cells prepared at 10 weeks after initial infection. **A** Flea-derived *w*CfeJ. **B** Sand fly-derived *w*Pap. **C** Mosquito-derived *w*AlbB passaged onto fresh GMA/LULS61 cells 5 weeks after initial infection. Scale bars = 10 µm; arrows indicate bacteria

Seven strains of *Wolbachia*, originating from mosquitoes, sand flies, fruit flies, butterflies, fleas and planthoppers, were tested for infectivity for the GMA/LULS61 cells. Three weeks after inoculation, *Wolbachia* strains *w*CfeJ and *w*Pap were present as low-level infections (< 1 in 100 cells infected in Giemsa-stained cytocentrifuge smears). At 10 weeks after inoculation, infection rates of both *w*CfeJ and *w*Pap had increased to around 50% (Fig. 6A, B); neither culture showed appreciable cytopathic effect (CPE) during the 13-week observation period. In contrast, by 3 weeks after infection, strains *w*AlbB and *w*Str1 had already caused heavy infections in GMA/LULS61 cell monolayers with accompanying CPE manifest as areas of detaching or dying cells. These two strains were subcultured onto fresh GMA/LULS61 cells at 5 weeks, and both caused high infection rates and CPE within a further 5 weeks (Fig. 6C). Cells inoculated with *w*Bol1-b and *w*MelPop-CLA were detectably infected at 3–4 weeks after inoculation and had achieved a high infection rate (> 50%) by 6 weeks; both strains caused CPE resulting in death of most of the host cells by the end of the observation period. Infected cells were not seen in two independent cultures inoculated with strain *w*Pip and observed for 11 and 24 weeks; both cultures were negative by *Wolbachia* 16S rRNA qPCR when tested at the end of the observation periods.

## Discussion

Insect cell lines play a vitally important role in many aspects of research on biology and control of insect vectors and the pathogens they transmit [21–23]. We anticipate that the new GMA/LULS61 cell line will at least partially fulfil this role in advancing knowledge of *G. m. morsitans* biology at the cellular, molecular and genomic levels and providing an environment for in vitro study of interactions among tsetse flies, viruses, intracellular and extracellular bacterial symbionts and trypanosomes. The only other tsetse cell line ever developed, the embryo-derived WR73-Gm-1, supported partial development in vitro of *T. congolense* bloodstream forms into procyclic and epimastigote forms but not development of infective metacyclic forms [5]. In contrast, transformation of procyclic forms of *Trypanosoma brucei* into metacyclic forms infective for mice was achieved through co-cultivation with adult *G. m. morsitans* tissue explants [24]. It will be interesting to see whether the adult-derived GMA/LULS61 cells provide an environment capable of supporting development of mammal-infective trypanosomes.

Amongst the > 1000 insect cell lines established over the past 63 years [8], most were derived from embryos, larvae or pupae, while ~ 40 cell lines were derived from adult tissues. Around three-quarters of these adult-derived insect cell lines are of ovarian origin, with a few each of testis, midgut or haemolymph/haemocyte origin [8]. It is unclear exactly which adult female tsetse tissues gave rise to the GMA/LULS61 cell line, but as they immediately surrounded larvae dissected out from the flies’ abdomens, it is likely that they were of ovarian origin.

The GMA/LULS61 cells grow relatively slowly compared to other insect cell lines; although we have not determined the doubling time, it is currently much longer than the doubling times reported for other adult-derived cell lines: 14.5 h reported for the *Trichoplusia ni* (cabbage looper moth) ovary-derived line TN-368 [25], 2 days for the *Drosophila melanogaster* female germ-line stem/ovarian somatic sheath line fGS/OSS [26] and 3 days for the *Sitophilus oryzae* (rice weevil) midgut-derived line SoMG [27]. Cell lines derived from embryos or larvae of other biting insects also tend to have short doubling times: 6–18 h for the *Anopheles gambiae* larva-derived line LSTM-AG-55 (Mos55) [28], ~ 14 h for the *Culicoides sonorensis* embryo-derived line KC [29], 24 h for the *Aedes aegypti* line Aag2 [30] and ~ 67 h for the embryo-derived *Haematobia irritans* embryo-derived line HIE-18 [31]. The *G. morsitans* larva-derived cell line WR73-Gm-1 was also reported to be very slow growing initially — the first subculture was carried out 4–5 months post initiation, and the growth rate did not increase until 10 months later; once established, the doubling time of the WR73-Gm-1 cells was 72 h at 24–26 °C [6]. It is possible that the growth rate of GMA/LULS61 cells will continue to improve over time and with increasing passage level; the reduction in successful subculture interval from 4 to 2 weeks observed at passage ~ 20 is encouraging.

Compared to the size reported for the WR73-Gm-1 cell line, most of the GMA/LULS61 cells are notably smaller. Dimensions of 10–20 µm wide and 15–30 µm long for WR73-Gm-1 cells were reported [6]; in contrast, the majority of GMA/LULS61 cells measured between 7.0 and 9.3 µm in diameter. This size difference could be due to different tissue origins of the two cell lines (third-stage larvae versus adult) and/or the relative unpredictability of arthropod cell culture, in which it is generally not possible to predict what cell phenotypes will start to grow in a primary culture. However, another notable difference between GMA/LULS61 and its predecessor is the number of chromosomes in each cell. Over 90% of WR73-Gm-1 cells were reported to harbour 8 chromosomes, with the remaining 10% of cells having more than 10 chromosomes, aligning with the previously reported diploid number of 8 or 10 ± 1 [6]. The two metaphase spreads illustrated in that report show four approximately metacentric autosomes and two sub-metacentric sex chromosomes per cell. In contrast, although the modal number of chromosomes in GMA/LULS61 cells at passage 17 was 8, most metaphase spreads contained only two large metacentric autosomes and a single sex chromosome, plus much smaller telocentric “S” chromosomes and/or microchromosomes. This observation raises the possibility that the majority of the cells in the GMA/LULS61 cell line could be haploid. Further studies are required to explore this potentially interesting phenomenon. Although rare (around 1% of insect cell lines, [8]), mosquito and *Drosophila* cell lines with at least partially haploid karyotypes have been reported previously [32–34].

As expected, molecular analysis of two genes, 18S rRNA and COI, confirmed the species origin of the GMA/LULS61 cells as *G. m. morsitans*. The cell line can therefore be applied to more in-depth studies of *G. m. morsitans* at the cellular, transcriptomic and genomic levels. These studies, in addition to providing insights into tsetse biology, are likely to reveal presence of any additional endogenous RNA or DNA viruses in the cells. As a preliminary investigation, we screened the cells for presence of three viruses known to infect *G. m. morsitans* both in the wild and from laboratory colonies.

At least three viruses have been associated with *G. m. morsitans*: SGHV, a double-stranded DNA virus in the family Hytrosaviridae causing hypertrophy of salivary glands in infected adult flies [35, 36], and two RNA viruses, the iflavirus GmmIV and the negevirus GmmNegeV, neither of which are known to cause any deleterious effect on infected flies [18], nor have they previously been isolated and propagated in cell culture. The GMA/LULS61 cell line was found to be persistently infected with GmmIV and GmmNegeV, but there was no evidence of infection with SGHV. To date, SGHV has not been propagated in vitro in any cell culture system; GMA/LULS61 cells offer the possibility of isolating and studying SGHV in cells derived from the natural host genus. The possibility that presence of GmmIV and GmmNegeV in tsetse flies might offer protection against SGHV infection has been suggested [18]; GMA/LULS61 cells offer a potentially useful in vitro system for investigating antagonistic and/or synergistic effects of these three viruses on host cell immunity through silencing of virus and/or host genes.

*Wolbachia* were first reported in ovarian tissue of *G. m. morsitans* obtained from a laboratory colony of unspecified geographic origin [3]. A subsequent study from the same group, but possibly using flies of a different population or geographic origin, reported prevalence of a supergroup A *Wolbachia* of 100% (10/10) in *G. m. morsitans* [37]. However, it is now known that the *G. m. morsitans* genome contains extensive lateral gene transfers from its *Wolbachia* symbiont [38], which can complicate the results of molecular screening. In the present study we did not detect any microscopic or molecular evidence of infection with *Wolbachia* bacteria in the GMA/LULS61 cell line, which corresponds with data obtained from whole tsetse flies from the same laboratory colony [39]. We therefore assessed the ability of the GMA/LULS61 cells to support infection and replication of a panel of *Wolbachia* strains originating from other dipteran (mosquitoes, sand fly, fruit fly), siphonapteran (cat flea), lepidopteran (butterfly) and hemipteran (planthopper) hosts. Most of the *Wolbachia* strains were able to infect and grow in the tsetse cells, with the exception of *w*Pip, derived from the mosquito *Culex pipiens molestus* [12]. However, there were considerable differences in the impact of the different *Wolbachia* strains on the GMA/LULS61 cells; *w*AlbB, *w*Bol1-b, *w*Str1 and *w*MelPop-CLA started to kill the host cells within 3–8 weeks, while heavy infections with *w*CfeJ and *w*Pap were tolerated without causing appreciable CPE throughout the 13-week observation period. These finding suggest that GMA/LULS61 cells could be used to isolate and propagate *Wolbachia* strains from *G. m. morsitans* and other *Glossina* spp., thereby facilitating their characterisation.

## Conclusions

The GMA/LULS61 cell line has potential for application in a wide range of studies investigating the biology of *G. m. morsitans*, and tsetse flies more generally, and their interactions with the viruses, bacteria and protozoa that they harbour and/or transmit. The cell line will provide a useful addition or alternative to working with live tsetse flies, thereby contributing to reduction in animal experimentation and allowing laboratories without access to the whole insects to contribute to tsetse research. The GMA/LULS61 cell line is deposited in, and available from, the Tick Cell Biobank at the University of Liverpool https://www.liverpool.ac.uk/research/facilities/tick-cell-biobank/.

### Supplementary Information

**Additional file 1: Figure S1.** Reverse transcriptase quantitative PCR (qRT-PCR) amplification and melting points of iflavirus, negevirus and tubulin (extraction control) detected in *Glossina morsitans morsitans* cell line GMA/LULS61. Blue = cell line; red = positive control; green = negative control. Amplification (left) is the correlation between the relative fluorescent units (RFU) and the number of cycles. The melting point (right) shows the correlation between the change in relative fluorescence over temperature (d(RFU)/dT) and the temperature. Measurements were taken in triplicate.

## Data Availability

All data generated or analysed in this study are included within the article.
