# Supplementary figures and images for: Establishment and partial characterisation of a new cell line derived from adult tissues of the tsetse fly Glossina morsitans morsitans
Source: Parasit Vectors. 2024 May 17;17:231. doi: 10.1186/s13071-024-06310-9 (PMC11100113; doi:10.1186/s13071-024-06310-9)

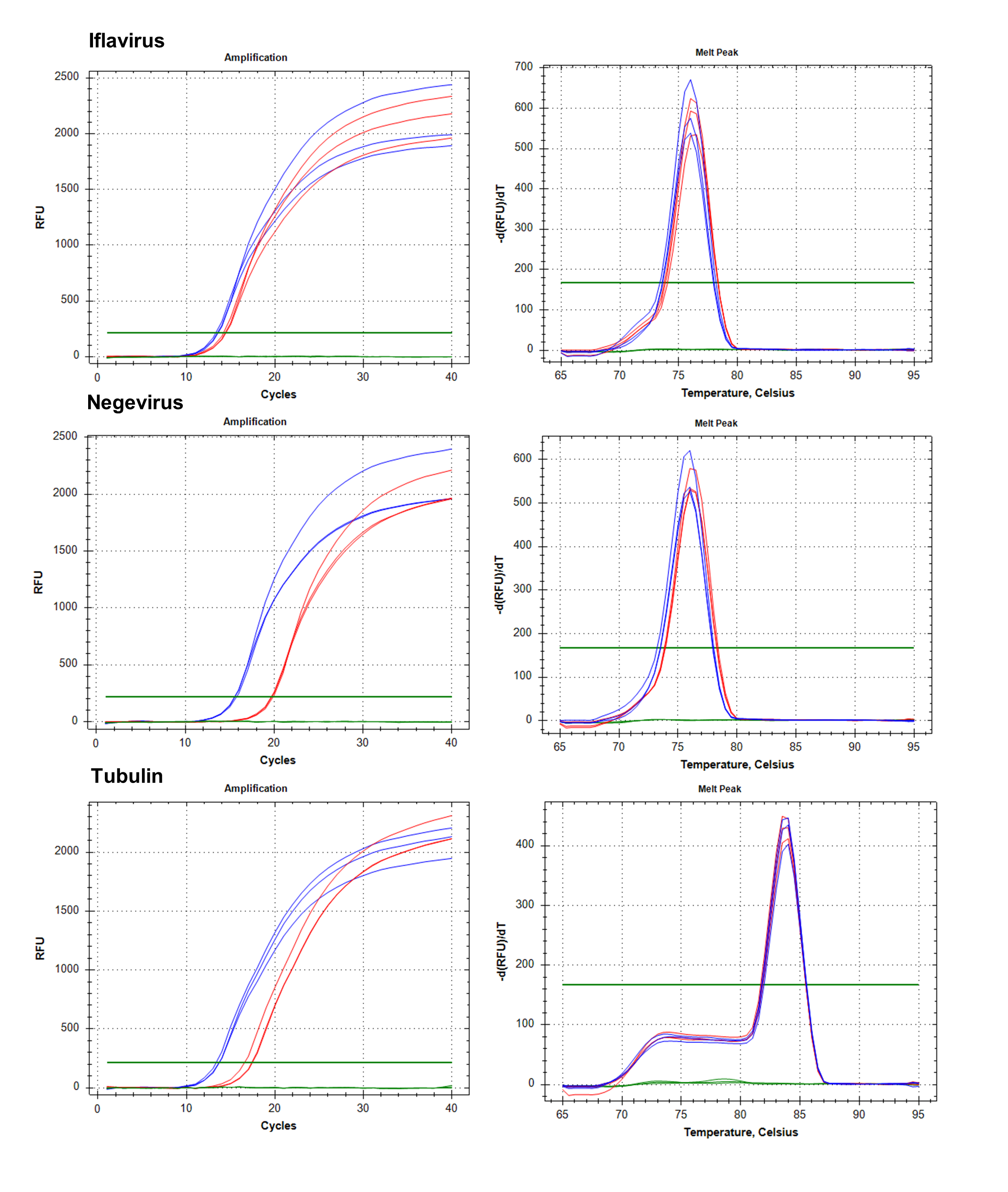

Supplement: Supplementary file 1 — Additional file 1: Figure S1. Reverse transcriptase quantitative PCR (qRT-PCR) amplification and melting points of iflavirus, negevirus and tubulin (extraction control) detected in Glossina morsitans morsitans cell line GMA/LULS61. Blue = cell line; red = positive control; green = negative control. Amplification (left) is the correlation between the relative fluorescent units (RFU) and the number of cycles. The melting point (right) shows the correlation between the change in relative fluorescence over temperature (d(RFU)/dT) and the temperature. Measurements were taken in triplicate. [file 13071_2024_6310_MOESM1_ESM.tif]
